# Operationalisation of person-centred care in a real-world setting: a case study with six embedded units

**DOI:** 10.1186/s12913-022-08516-y

**Published:** 2022-09-14

**Authors:** Helena Fridberg, Lars Wallin, Malin Tistad

**Affiliations:** 1 School of Health and Welfare, Dalarna University, Falun, Sweden; 2 Institute of Health and Care Sciences and University of Gothenburg Centre for Person-Centred Care, Sahlgrenska Academy at the University of Gothenburg, Gothenburg, Sweden; 3 Department of Neurobiology, Care Sciences and Society, Karolinska Institute, Stockholm, Sweden

**Keywords:** Implementation, Person-centred care, Case study, Operationalisation, Core components

## Abstract

**Background:**

Although person-centred care (PCC) is growing globally in popularity it is often vague and lacks conceptual clarity and definition. The ambiguity stretches from PCC’s underlying philosophical principles and definitions of the concept to how it is operationalised and practised on the ground by health care professionals. We explore how the PCC model by the Gothenburg University Centre for Person-centred Care (GPCC) was operationalised in a real-world setting by using a set of recommendations by Fixsen and others that define and structure the core components of innovations in four distinct but interrelated components: philosophical principles and values, contextual factors, structural elements and core practices. Thus, this study aimed to increase knowledge about core practices in PCC in six health care units in real-world circumstances.

**Methods:**

A case study with six embedded health care units was conducted from 2016 to 2019. We collected data from three sources: interviews (*n* = 12) with change agents, activity logs and written documents. Data were triangulated, and core practices were identified and deductively coded to the PCC model’s structural elements: initiating, working and safeguarding the partnership with patients.

**Results:**

We identified operationalisations of PCC in line with the three structural elements in the GPCC model at all included health care units. A range of both similarities and dissimilarities between units were identified, including the level of detail in describing PCC practices, when these practices were conducted and by whom at the workplace. The recommendations for describing the core components of PCC also helped us identify how some operationalisations of PCC seemed more driven by contextual factors, including a new regulation for planning and documenting care across health care specialities.

**Conclusions:**

Our findings show how PCC is operationalised in different health care units in a real-world setting based on change agents’ understanding of the concept and their unique context. Increased knowledge of PCC and its philosophical principles and values, contextual factors, structural elements and core practices, is necessary to build a common understanding of the PCC-concept. Such knowledge is essential when PCC is operationalised as part of implementation efforts in health care.

**Supplementary Information:**

The online version contains supplementary material available at 10.1186/s12913-022-08516-y.

## Background

Person-centred care (PCC) is a concept that is gaining ground among policymakers, managers, health care professionals (HCPs) and patient organisations worldwide [1–3]. PCC relies on philosophical and ethical principles of “seeing the person behind the patient” [4, 5]. This paradigm shift creates a transition from paternalistic care based on HCPs focusing mainly on medical parameters and viewing the patient as a passive receiver of health care to sharing power, information and decisions about health care in partnership with patients [5, 6]. Because care is co-created in partnership, each meeting will become unique, relying on peoples’ interactions and communication skills, often in situations of high levels of personal autonomy, i.e., relying on each HCPs and patients degree of adopting a PCC approach [5, 7, 8]. The concept of PCC is not only related to the individual meeting between HCPs and patients but is also acknowledged to include and engage the whole health care sector, with different stakeholders at different levels within and across organisations to take on the challenges and transition towards delivering more PCC [1, 9, 10].

PCC has an inherent complexity in that there is a natural variation in the ways it is operationalised. Consequently, what practices PCC entails will depend on patient needs and the context in which care is undertaken [4, 5]. Self-management support [11], personalised care planning [6, 12] and shared decision making [13] are examples of approaches to operationalising and implementing PCC in practice. One dilemma for those struggling to implement PCC in a real-world context is the lack of consensus in the literature regarding defining, conceptualising, operationalising and measuring PCC [4, 10, 14–16]. PCC and similar concepts (e.g., patient-centred care and patient-centred medicine) originate from different schools of thought with a lack of common terminology and conceptual clarity [2, 4, 5, 10, 17, 18]. To complicate matters, published studies on how PCC is operationalised in interventions often lack details and clarity about PCC’s theoretical, empirical or ethical principles [1, 9, 19]. In addition, efficacy studies on PCC often report outcomes of an intervention but without reference to what or which of the intervention’s components entailed the PCC activity(s) that contributed to the outcome [15]. Poor descriptions of the operationalisation of an abstract concept (such as PCC) precludes replications and generalisations in the research community and hinders guidance of what works for whom in clinical practice [15]. Proponents of PCC reason that to succeed with its implementation, the fundamental ethical principles need to be embedded and understood by the HCPs working in everyday PCC activities [8, 9]. However, to evaluate whether implementation is successful and what activities within an intervention represent PCC, researchers need to state what these activities are [20]. With detailed descriptions of the intervention, comparisons across studies can be made and an increased understanding achieved built on the situations in care that have been successfully transformed to be consistent with teachings of PCC. PCC as a new practice is referred to as *innovation* in this study [21].

To understand how innovations are operationalised and thought to work, logic models or programme theory have been advocated [22]. However, these models and theories often lack a clear connection to how contextual factors may affect the operationalisation of the innovation [22]. The lack of description of activities that establish PCC on the level of health care practice may lead to difficulties to reach agreement on to what extent the practice already is person-centred and to achieve consistency in practice across practitioners, departments and units [23]. Fixsen et al. have presented criteria for how innovations can be specified and described to be taught, learned and implemented with fidelity [24, 25]. According to these criteria, a well operationalised and defined innovation includes a description of the philosophical principles and values that underpin the innovation, contextual factors, the core component in terms of structural elements and operational definitions of these structural elements that allow the innovation to be doable in health care practice [24, 25]. One PCC model widely used in Sweden [6] with considerable influence on the content of the European SIS Standard for PCC [3] is the Gothenburg University Centre for person-centred care (GPCC) model [5, 6]. The GPCC model for PCC departs from the philosophical and ethical underpinnings of person centredness which have been further developed to include three routines aimed at guiding HCPs in understanding how PCC can be operationalised in practice. These routines are based on developing and sustaining a partnership between HCPs and patients through three related activities: initiating, working, and documenting the partnership [5, 6]. Operationalisations of PCC in line with the GPCC model have been used in various research projects with different activities and foci based on factors such as type of care environment, patient needs, and staff involved [26]. Examples of operationalisations include practices such as person-centred telephone-support for patients with chronic obstructive pulmonary disease and chronic heart failure [27], the introduction of written and verbal person-centred communication tools for patients with colorectal cancer [28], and tailored physical activity for patients with rheumatoid arthritis [29]. Inspired by Fixsen et al., we will investigate the implementation of PCC based on the GPCC model in a Swedish health care context to identify operational definitions of PCC. Thus, this study aims to increase the knowledge about core practices in health care constituting PCC in a real-world setting.

## Methods

This case study is part of the IMPROVE project (Implementing person-centred care, process evaluation of strategies, leadership and health economics) [30, 31], in which the implementation of PCC in a health care region in Sweden was explored between 2016 and 2019. A variation of participants (e.g., patients, HCPs, managers, politicians) and data sources (e.g., interviews, surveys, logbooks, health care records) at multiple levels within the organisation have been used to give a holistic view of the region’s implementation efforts.

The larger project includes studies such as HCPs perceptions of PCCs innovation characteristics [23] and patients’ perceptions of PCC through the development of a questionnaire [8]. The IMPROVE project is a case study with seven embedded units and in this study six units (those delivering health care to patients) were included. A unit has been defined for the purpose of the project as a medical or administrative facility specifically staffed, equipped and organised to provide particular health care or support functions in the health care region.

### The case

The case is defined as the *operationalisation of PCC.* The case study approach was considered suitable because the operationalisation of PCC was conducted as part of current implementation efforts without involvement from researchers in a real-world setting represented by six units from various health care specialities.

### Setting

This study took place in a region in central Sweden with approximately 280,000 persons in an area of 28.000 km^2^. Inhabitants in the area were provided with health care by one large regional hospital, five local hospitals and about 30 primary health care units.

In 2015, the regional political assembly adopted a policy regarding “increased participation in the health care services for patients, relatives and patient and user organisations”. The policy was embodied in PCC and in accordance with the conceptualisation of PCC by GPCC [6].

At the regional level, staff at the Department for Development (DD) were assigned to develop a strategy to support the change to more PCC across the region. The support strategy included an offer to all health care units in the region to participate in a series of three full-day learning seminars intending to disseminate knowledge about PCC and provide initial support in their implementation work. The staff at the DD decided to introduce PCC through GPCCs model for PCC. The senior and frontline managers at each health care unit decided who and how many HCPs should participate in the seminars. They were strongly encouraged to enrol a wide selection of HCPs to enhance team discussions. The learning seminars included lectures, workshops and discussions run by researchers from GPCC, people from other health care regions in Sweden, patient representatives and health care staff from the units in the region. An important feature of the DD’s support strategy was to regard change agents, i.e., those responsible for implementing PCC at the health care units, as autonomous and inheriting unique knowledge about their context. Change agents were trusted to use their understanding of patients and their specific needs, HCPs and work routines to decide how they wanted to operationalise and implement PCC. Another strategy used to support the implementation of more PCC by the DD in the region was introducing the search word - *the narrative* - and developing a template for health care plans in electronic health care records.

### Framework for operationalisation of PCC core components

**Fig. 1:**
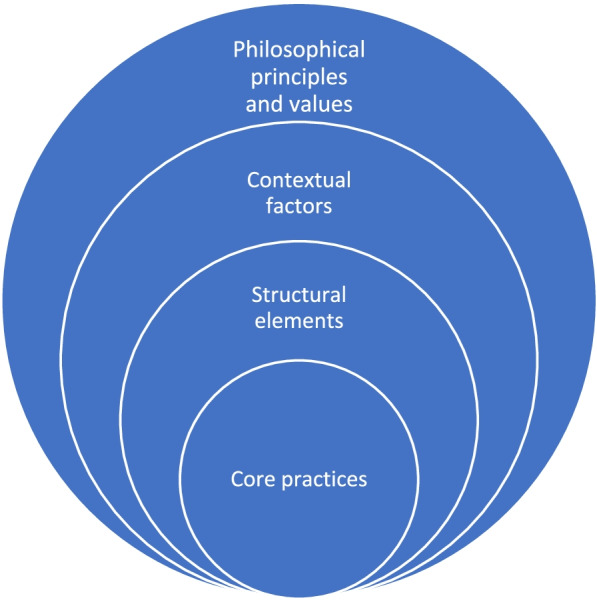
The core components of an innovation adapted from recommendations by Blase and Fixsen

According to Blase and Fixsen et al. [21, 22], core components are the essential functions of an innovation necessary to achieve the expected outcome. Core components can be established as philosophically, theory-driven or empirically derived principles and then further operationalised as intervention practices aligned with the principles. The components that define a well-operationalised innovation, adapted to this study’s purpose, are depicted in Fig. 1.

### The core components – from principles to practices

The three higher-order elements, i.e., the philosophical principles and values that underlie the innovation, the context of the innovation and the structural elements that define the innovation, are applied to PCC according to the GPCC model. The GPCC model was used in this study as it was the model introduced and chosen by representatives in the region. The higher-order elements are described below and lay the foundation for the focus of this study, which is to explore and describe the core practices that constituted PCC in six health care units.

#### Philosophical principles and values

Philosophical principles and values that underpin an innovation should be well described to support consistency in practice and provide guidance in innovation-related decisions [24, 25].

In the GPCCs model of PCC the fundamental principles are built by an ethical approach based on person philosophy [5, 6, 32]. An individual takes on the role of a patient when introduced to the health care sector. Seeing the person behind the patient becomes an important aspect as it takes a broader perspective of the person’s life than just their disease or illness. Every person is seen as unique with individual wishes, resources, experiences and goals, all of which need to be considered to co-create care that seeks a meaningful life for each patient [17].

#### Contextual factors

Contextual factors include the environment where the innovation is used and the population targeted by the innovation [24, 25]. The larger context for PCC is the Swedish healthcare system organised in 21 regions throughout the country and publicly funded by taxes. Each region has considerable freedom to manage their health care mission provided that they stay within Swedish legal limits. Most health care regions in Sweden have decided to implement more PCC [33, 34].

The population targeted by the innovation is all patients in Sweden. The population relates directly to the ethical principles and values of PCC advocating a health care sector built on standards of equality for all people in society [5]. Implementation of PCC in Sweden was strengthened when the Swedish Patient Act [35] was introduced in January 2015. The law was created to reinforce patients standing in the Swedish health system and points at concepts similar to those advanced in teachings on PCC, stating, for example, that patients have a right to be informed about their illness and treatment options, and take an active part in discussions concerning their health care. Another law [36] was introduced in 2018, stipulating that patients in inpatient care and in need of continued support from other stakeholders upon discharge should have an individual health care plan containing information about the continued care or support needed [36]. Moreover, health care records in Sweden are written and stored in electronic databases. Patients have the right to read their records via electronically protected platforms, to varying degrees.

The specific context and population related to each health care unit are described in the participant section.

#### Structural elements that define the innovation

The structural elements, also denoted as the essential functions, must be present to notify that an innovation exists in a setting [24, 25]. In terms of GPCCs’ model of PCC the three clinical routines thought to be in line with the ethical aspects of PCC are considered structural elements in this study [5, 6]. These routines are promoted to support HCPs to work according to PCC in different contexts. First, they initiate a partnership by listening to patients’ narratives to understand their resources, abilities, personal priorities and values about their health and illness and the factors that matter in their lives. Second, working the partnership by discussing and sharing information to co-create care and make decisions about health and personal welfare (e.g., medical investigations, treatments and self-management). This second routine ends with a commonly agreed upon plan for action, including decisions and continued care and treatment goals. Third, safeguarding the partnership is achieved by documenting the plan. The plan should be revisited regularly and revised according to discussions between HCPs and patients [5, 6].

#### Core practices

Core practices are operationalisations of the central components that make those components feasible in a health care sector and need to be specified well enough to be teachable and learnable [24, 25]. The focus of this study is the core practices, which are described in the result section in relation to the structural elements in the GPCC model.

### Participants

We recruited a convenience sample of six health care units in the region, representing care from a broad set of patient needs and contextual characteristics via email. The sample was based on the number of units (*n* = 11) that participated in the first PCC teaching initiative in the region, aiming to represent a diversity of care, and with senior and frontline managers consenting to participate in the study. The six recruited health care units had contexts related to various factors. These factors included location in the region, sector within health care (e.g., in- or outpatient care), type of care, patient characteristics and patients need for care. Patient care could range from a single visit for a health check-up in primary care to haemodialysis at the nephrology unit three times a week all year round, and to inpatient care entailing care around the clock up to a median stay of 23 days (see Table 1 for a description of the health care units’ contextual factors). The health care units also showed a large diversity in HCP characteristics related to occupational roles and number of employees.

**Table 1:**
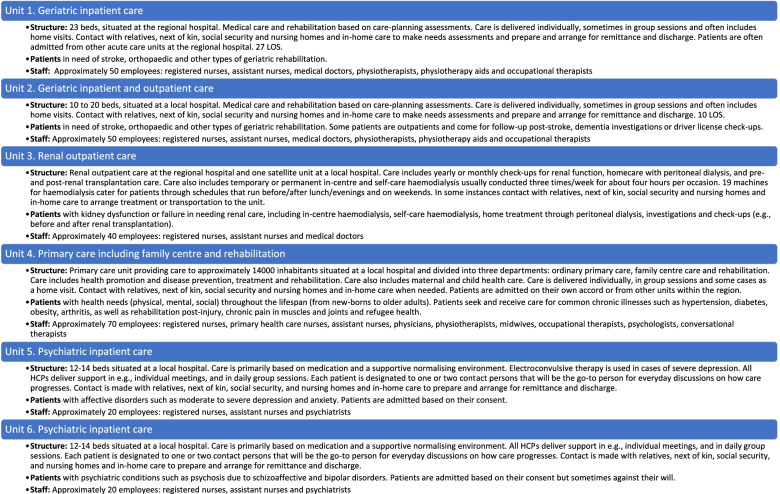
Contextual description at the unit level, including structure and patient and HCP characteristics

Nb. Units 5 and 6 were merged into one ward due to a staff shortage between June 2016 and August 2018, with 18 beds available in the merged ward during this period. Medium LOS was 15 days, including temporary leave

*LOS* Length of stay

We recruited 24 staff (4 men) – hereafter referred to as *change agents* – in charge of the implementation at each of the six health care units. The number of participants in each interview was based on staff members regarded as change agents at each unit by the managers. Dyadic interviews (DI) [37] were conducted for units 1 and 2, whereas staff from units 3 to 6 participated in focus groups (FG) [38] as shown in Table 2.

**Table 2:** Number of change agents participating in interviews at each health care unit and their occupational roles

Unit	n	Occupational role
1	2	Frontline manager Assistant frontline manager
2	2	Frontline manager Quality developer
3	6	Frontline manager Assistant frontline manager HCPs represented by different care specialities at the unit: nephrology investigations and check-ups, haemo-, peritoneal and home dialysis.
4	6	Senior manager Quality developer Frontline managers representing three departments: Ordinary primary care, family centre and rehabilitation
5	4	Frontline manager Coordination nurse Registered nurse Assistant nurse
6	4	Frontline manager Coordination nurse Registered nurse Assistant nurse

### Data sources

Data were collected from three sources: activity logs, interviews and written documents (e.g., reports and presentation plans at learning seminars).

#### Interviews

The interviews and focus groups took place on a date, time and location designated by the frontline manager at each unit. All interviews were conducted in secluded rooms close to each unit’s workplace. Interviews, lasting from 41 to 98 (mean 65) minutes, were conducted twice at each health care unit approximately a year apart. The first and last authors, both with experience at interviewing, took turns acting as moderators/interviewers. These two authors are female, have a background in physiotherapy, and have not worked at any of the included units. Further information about the reflexivity characteristics of the authors is found under the heading *Authors information*. All authors were engaged as notetakers. A semi-structured interview protocol was used to explore how the operationalisation of PCC had been realised at the different health care units (Additional file 1) [39]. The interview protocol also contained questions with another aim: to explore which and how strategies had been used to support implementation of PCC. Data exploring the strategies will be reported in a separate article. Prompts were used to analyse comments perceived by the moderator to lack detail (e.g., participants describing that they perceived patients as capable and resourceful) [40]. They were then asked how this was achieved in actual practise at the unit. The moderator/interviewer used ongoing summaries of participants’ comments and discussions to ensure they interpreted what was being said correctly, i.e., member validation in real time [40]. Moreover, an overview was made of the information shared by the participants after the first round of interviews. The participants were asked to validate these data at the second round of interviews. Interviews were recorded and transcribed verbatim.

#### Activity logs

We developed an electronic activity log in line with previous work and recommendations by Bunger et al. [41]. Change agents were assigned to use this log to report all implementation activities enacted to achieve more PCC [41, 42] during the study period. Reports focused on describing the activity being performed to support the implementation. Details on these logbooks will be provided elsewhere in a study focusing on implementation strategies. Only clarifying comments from the change agents who kept the activity logs [43] were used for this study.

#### Documents

We collected documents at the health care unit level that we interpreted as related to activities passed to operationalise PCC at the units. Documents included guidelines targeting HCPs, plans for educational meetings, workshops and reports at the unit level.

### Data analysis

Analysis was conducted separately for each unit to describe the core practices. Data from interviews, activity logs and documents were imported and analysed in NVivo. All data interpreted as descriptions of operationalisations of PCC were identified and then deductively coded to the GPCC model, i.e., structural elements. Data from interviews, activity logs and documents were triangulated to obtain the most credible analysis and interpretation of the data.

The analysis was performed by the first author and reviewed and discussed weekly in close collaboration with the co-authors.

## Results

Operationalisation of PCC at the health care units shared several similarities and dissimilarities, shown in Table 3 and further elaborated in the text and with quotes below. All units are represented by different quotes in the text.

**Table 3:** Operationalisation of PCC into core practices at the six health care units

Unit	Initiating the partnership	Working the partnership	Safeguarding the partnership
1	New admission routine where assistant nurses ask patients questions in a standardised protocol about their life before admission to get a deeper understanding of each person’s needs and wishes for care. Conversation methodology in line with MI are encouraged (coordination nurses) to listen closely to patients’ narrative as well as enable discussions when they are working the partnership. The narrative is explored by a range of HCPs during patients stay at the ward	Patients are encouraged to be more in charge of their rehabilitation process by setting goals in line with their own wishes and needs and be part of taking decisions about their care. Goals are discussed and revised between the patient and HCPs on a weekly basis. Coordination nurses at the ward take extra responsibility of rehab plans as an ongoing process where plans for discharge is initiated on admission to the ward and changed at regular intervals. These plans are developed together with the patient and all HCPs involved in their care. Everyday care routines such as eating habits or showers on a weekly basis are encouraged to become more flexible according to patients wishes i.e., showers twice a week. Increased partnership is enabled through the introduction of videoconference equipment. Patients, their next of kin and other stakeholders e.g., at nursing homes can meet to discuss and plan for discharge, continued care and rehabilitation.	Patients’ narratives are documented in the health care record. Rehab plans are documented in the health care record.
2	Conversation methodology in line with MI are encouraged (for HCPs working in outpatient rehabilitation) to aid HCPs to listen closely to patients’ narrative as well as enable discussions when they are working the partnership.	Patients are invited to co-create their care in team meetings with HCPs where ongoing-, planned care and rehabilitation are discussed. Rehab plans are developed in partnership including short- and long-term goals based on each patient’s wishes and needs. Patients are given access to training facilities around the clock at the ward as they are viewed as resourceful and capable. Introduction of home rehabilitation enables an improved discharge process where rehab in the ward is followed through to the patients’ homes and guided by patient wishes and needs in relation to their home environment. New treatment alternatives are introduced to increase choices for rehabilitation for patients e.g., horse rehabilitation.	Rehab plans are documented in the health care record.
3	Patients’ narratives are elicited on admission. Warm handovers for patients transitioning between care specialities using patients’ narratives. Conversation methodology in line with MI are encouraged to aid HCPs to listen closely to patients’ narrative as well as enable discussions when they are working the partnership.	Increased work in partnership with all patients to explore their resources and wishes for increased responsibility in self-care. A self-care document is used where patients are asked if they wish to learn more and take more responsibility for self-care, participation and support in care activities which is documented into concrete activities. Examples of activities are patients doing their own calculations on how much fluid to pull in conjunction to dialysis. Starting up a teaching program for new patients to increase patients’ knowledge on kidney disease, diet, and dialysis modalities. Improve patients’ feelings of safety and freedom and using own resources by introducing videoconference equipment.	Patients’ narratives are documented in their own words under the heading narrative in the health care record. Patients’ wishes and agreements for care are documented.
4	Patients’ narratives are elicited on admission. HCPs are encouraged to listen to patient wishes. Conversation methodology in line with MI are encouraged to aid HCPs to listen closely to patients’ narrative as well as enable discussions when they are working the partnership.	Increased teamwork in partnership with several HCPs and the patient to meet patients’ needs and wishes, discuss goals and treatment plans and improve transitions between different HCPs within the unit. Patients are given information and support in line with their wishes such as being asked to describe how and what kind of information they would like, and how and when they would like to be contacted for follow up. Improve information to patients about treatment alternatives to ameliorate participation in decisions. Starting up a lifestyle unit catering for patients who are not ill but is running a risk of getting ill. The lifestyle unit is based on patients seen as resourceful and capable to use information, support and guidance to make lifestyle changes and make their own choices of how they want to live their lives.	Patients’ narratives are documented in the health care record using the search word narrative.
5/6	Patients’ narratives are sought on admission. Conversation methodology in line with open dialogue are encouraged.	Changing the daily round to make more time talking directly with the patient compared to talking about the patient. Defining the role of the contact person to fit closer with PCC, involving taking the time to listen closely to patients’ narratives, discuss goal setting, follow up on goals, weekly plans, wishes for care and if there are issues that needs to be raised in meetings with psychiatrists, social security or at home. Activity plans and goals that patients make in group sessions are followed up by each patients contact person and discussed and revised in relation to individual needs, resources and goals. The contact person participates and support the patient at meetings with psychiatrists and other stakeholders such as social security personnel.	A health plan based on the patient’s narrative, including a planned remittal date is written together with the patient on admission and revised regularly.

Nb. Units 5 and 6 were merged during a large part of the study period. They tried to stay with their original teams and work with patients with familiar diagnoses. However, operationalisation of PCC was developed in one of the units and then transferred to the other unit resulting in operationalising PCC with the same take in the end

### Overall aspects of the operationalisations

Change agents could to various degrees elaborate on their thoughts about how the operationalisation of PCC had been accomplished through its ethical underpinnings. They traced their descriptions in a back-and-forth process between PCC’s ethical underpinnings, their contexts, PCC’s structural elements and concrete practices at their units during discussions and when prompted by the moderator to do so. Change agents were sometimes able to explain how a specific activity was connected to PCC or how an abstract construct (such as the mentioning of increased participation) transferred to concrete practices at their unit. A change agent described how increased participation was fulfilled through creating rehab plans with patients.

*We started to do rehab plans together with the patients. We have not had a well-functioning routine of making rehab plans with the patients, and we saw the potential of working mutually and creating it together with the patients. The patients will be more involved in their care, set goals and [describe] what they want. You would think it would already be a natural part for us to work with rehab plans where the patient participates. But it has not been evident for us. So that’s our goal, to make the patients more involved in their rehabilitation.* (DI)

However, there were also instances when change agents were vague in their descriptions of how a specific practice could be traced back to its ethical basis or vice versa. Change agents at one unit described introducing a high-calorie diet for all patients, but they could not relate this change to the ethical foundation of PCC. Other change agents discussed how they encouraged HCPs to listen to, collaborate with and co-create health care with patients by using a flexible attitude towards patients’ wishes and values. Thus, change agents sometimes advocated a PCC approach without specifying how this could be attained or exemplified in specific practices at the workplace. Descriptions of the number of times an activity was advocated, its duration and when it should be carried out differed considerably between the structural elements targeted and the health care units. For instance, PCC core practices specified for point in time and number of occasions at the units included developing health care plans with patients on admission and discharge to enable the improved transition to other care specialities, social security and care at the municipality. Other practices where the point in time was specified were listening to patients’ narratives on admission. Moreover, HCPs at units 5 and 6 underwent a changed routine of the daily round. This new routine entailed changing how work was scheduled at the unit in conjunction with work tasks and new staff roles. Assistant nurses were charged to take on more responsibility as contact persons towards patients and other stakeholders related to the patient’s wellbeing. Such additional responsibility included involvement with staff at the health unit (i.e., psychiatrists and registered nurses), next of kin and social security. The new round aimed to have HCPs spend more time (increased duration) *talking to* patients and less time spent in conference rooms *talking about* patients. When this new routine was implemented, HCPs were given guidance and recall notes about posing open-ended questions and suggestions for topics and queries to enhance communication with patients.

PCC practices at the units sometimes relied on one HCP working in partnership with patients or were based on a whole team of HCPs working with patients to reach common goals and arrange care in line with patients’ priorities. Unit 4, in particular, and the other units represented by outpatient care, in which one HCP was primarily responsible for meeting a patient in individual meetings, had many of these one-on-one meetings between a HCP and a patient. In units where care was arranged in teams with HCPs with a variation of occupational roles and a coordinating nurse as the team’s hub, some PCC practices were instigated and completed by a collaboration of HCPs working jointly to remind and encourage each other to co-create care.

### Operationalisations of initiating the partnership

Listening to patients’ narratives was operationalised differently depending on how change agents perceived this aspect of PCC. Some change agents used in-depth descriptions of listening to patient narratives to understand their life experience, values and health care wishes. They also described that trust between the HCPs and patients had to be established for the patients to open and expose their true feelings. One change agent expressed how listening to narratives demands more from HCPs than just going in to say ‘hello,’ but what this “more” entailed was not clearly articulated.

*To talk with the patient and try to hear what she is thinking. Does she still have delusions? How is it with her suicidal thoughts? You can’t just see that when you go in and say good morning or if you take her blood pressure. You need to do something more. And I believe that patients need to trust you if they are willing to tell you something. (FG)*

Other change agents described how they operationalised narratives by using a set protocol to collect information about patients on admission to identify patient needs and other matters (e.g., if they were at risk of falling or had nutritional problems). However, the narrative was also expressed as something that needed to be explored daily or at each new visit to the health care unit as an ongoing routine. One change agent told how patients’ resources and needs could change daily and even during the same day. Thus, HCPs had to become attentive and listen to patients on all these occasions in health care:

*Well, if I go in and see Margret and say hello, I need to ask what are you doing here and why do you think I am here for? What do you need help with today? Instead of getting a rapport from a nurse who says she needs help with her hygiene. And then you go in and do it, and perhaps it was correct yesterday, but it becomes a truth also today because we keep on doing it. So, I believe that it is vital that HCPs embrace the thought of asking patients, right here and today, what do you need help with right now. (DI)*

Some change agents described how patients’ narratives were built over time. These accumulated narratives were sometimes due to the patients’ health status and when they could express a description independently. In other instances, the narratives were accumulated with the whole team’s help working with the patient. HCPs with different occupational roles listened to patients’ narratives from their perspective and added ongoing information to build a holistic account of their lifeworld.

### Operationalisations of *working the partnership*

The partnership was discussed and operationalised from different work routines at the health care units. For some change agents, working in collaboration with patients was already regarded as a natural part of everyday work before their introduction to PCC. For example, change agents working with patients in home dialysis described how working in partnership was a prerequisite for their speciality. Others expressed how they had started to inform patients about different aspects of their care so patients could make more informed decisions. Telling patients about available treatments, information about the illness and healthful choices along with difficult subjects such as giving information and respond to thoughts about shifting focus of care for patients nearing death was described to occur more often at the units. Some change agents discussed the legal contextual factors as having a strong bearing on how PCC was operationalised at their unit. One change agent described it as follows:

*Well, we got this new patient law, and there we have obligations to relate to. We have laws that stipulate that we should inform the patients. They should be able to decide some … I mean, what kind of care suits me. Do I want surgery or not? You need to be able to participate in your care. (FG)*

Change agents at all units used different communication skills to operationalise PCC. Motivational interviewing (MI) [44] was the most common communication methodology used by the units to aid HCPs to be attentive to patients’ narratives and work in partnership to co-create care. Some units decided that all HCPs should learn this methodology, whereas others felt that only the coordination nurses needed these communication skills. One change agent described the need to operationalise PCC in communication proficiencies.

*When you work according to MI, you are very explorative. I'll meet you where you are. What are your thoughts? Why do you think this is so? What do you want to do? And this is a person-centred approach. I [the patient] become more involved in my care and make my own decisions.* (FG)

Another change agent described how she saw MI as a tool to aid operationalisation of most aspects of PCC and expressed an attitude of great relief of using this communication methodology concerning constructs embedded in a PCC approach.

*One could say that I am entirely sold on MI. You can use it to explore resources and get patients more involved in their care. Instead of having this monologue, we can have a proper conversation where the patient is extremely engaged. (FG)*

Working in partnership and seeing each patient as unique differed greatly depending on each unit’s contextual conditions and prerequisites. In unit 1 changes about daily and weekly work routines at the ward to align with patients’ wishes regarding how often they could have showers, rise in the morning and take their meals were advocated as examples and means to operationalise the partnership. In unit 4, representing primary care, co-operation was promoted in encounters with patients and regarded as sharing information and establishing goals based on patients’ needs and not on convenience. Increased co-operation was also encouraged in routines related to patients staying in touch with HCPs at the unit. Increased flexibility by moving away from routinised ways of organising care following set rules as to follow up, check-ups and which HCPs to contact was promoted to meet patients’ wishes and needs.

*If I [the patient] feel confident and independent, I may want to book my own time using the net. I want to get my results on the net and log in and read them myself. Maybe I don’t want to have that much to do with us; instead, I manage most of it myself. But if I feel fragile, uncertain and insecure, I may need to have an established contact in primary care. I need to have someone that I can call when I crumble. We need to have many different ways to get in touch with us based on each person. (FG)*

Changing how care was organised at the environmental level to meet the underlying ethical principles of PCC and the structural element partnership were also realised at the study units This change occurred in unit 2, where patients were regarded as resourceful and capable and encouraged to use training facilities around the clock and on weekends when rehab personnel were unavailable. Other changes in unit 2 to meet patient expectations were the introduction of horse therapy and the possibility for home rehabilitation.

*Those who are medically stable but still in need of rehabilitation can have their rehabilitation in their home instead of the ward. Patients will continue their rehabilitation where it will be most beneficial for them. It will be fruitful for patients and our unit-- well, for everybody. We will have yet another choice [for patients]. We have inpatient and outpatient rehabilitation, and now we will also have home rehabilitation to tailor more to your [the patients] specific needs. (DI)*

Other examples of operationalising PCC through increased partnership were changes in how care was organised, such as introducing video conference equipment to enable patients from their homes to contact HCPs and for next of kin or other stakeholders to participate in team meetings with patients in inpatient care.

Operationalising PCC through an increased partnership was sometimes related to a perceived increased efficiency in care whereby listening to patients’ goals would make them more inclined to work harder to reach these goals instead of goals set up by HCPs. Below is one change agent’s description of this shift towards a perception of more efficient care.

*Well, what did the patient actually want? I cannot answer that question if I have not explored it. That’s where we have person-centredness. There is no idea for me to run my race because it will not get us anywhere. It’s not going to be efficient. In 2 months we will not see any change. So, in my opinion there’s where you have person-centredness. (FG)*

Working towards patients’ goals and aspirations was a commonplace discussion at all health care units. Goals were often discussed with patients and documented in a health care plan. Some units were explicit about following up on goals and made ongoing revisions on daily or weekly visits. In contrast, other units were less precise about how often goals were revisited and revised. While change agents at one unit described that they had already worked with rehab plans before they were introduced to PCC, they now saw that the introduction of PCC and working in a partnership meant that its operationalisation in a health care plan provided a new perspective. This new outlook implied that patients had become more involved throughout their care and rehabilitation process.

### Operationalisation of safeguarding the partnership

All units worked with documentation of plans made in co-operation with patients. The new law about plans that had to be used when patients transitioned from one care unit to another was often considered an important determinant for realising this part of PCC.

One change agent described operationalisation concerning documentation as follows:

*It becomes more like the patient tells the story because when we write [in the health care record], we are supposed to use search words, and it [the narrative] becomes quite chopped up. It is really nice to write a patient narrative and get it, how the patients tell their stories. (FG)*

Another change agent described how PCC was operationalised in a health care plan and revised according to the patient’s status.

*The doctors write a health care plan directly when patients are admitted based on the patients’ narratives. So, the whole [plan] is person-centred. And then, based on the narrative, a joint decision is taken together with the patient of how long you [the patient] need to stay with us. From there, we plan a discharge date … then, you need to revisit it [the plan] and see if things have become more complex than they were initially, then the length of stay will be longer. We are overall more person-centred along the whole way now. (FG)*

Change agents at the primary care unit and the specialised outpatient care unit discussed documentation of PCC achieved in a health care plan less in terms of legal aspects and more related to documentation of the narrative and commonly agreed goals. Documentation was regarded as a means for follow-up and enabling other HCPs to be involved in the patient’s future care.

## Discussion

We conducted a case study to increase the knowledge of how PCC can be operationalised into something doable by exploring the implementation process in six diverse health care units in a real-world setting. To improve our understanding and reporting of the operationalisation of the core components we used the structure recommended by Fixsen et al. [24, 25]. These recommendations were chosen to guide data analysis and the reporting of our results because of their strong linkage to contextual factors considered a key determinant in PCC practice [45].

Our results show that the operationalisations of PCC were sometimes similar between the six health care units and sometimes specific to individual units. These results are in line with previous research in which PCC is operationalised into different practices in the health care sector [6, 9, 26]. These findings were expected because of the units’ varied contexts and PCC’s unique features based on co-creating care consistent with patients’ preferences along with treatment alternatives and available resources.

The three structural elements from the GPCC model were identified in all health care units. This finding is similar to a study describing 27 intervention studies targeting PCC, of which 22 contained all structural elements from the GPCC model [26]. However, these interventions were planned and orchestrated by researchers tied to GPCC and not based on naturalistic studies [26]. Despite the identification and confirmation of the three structural elements at all units, the operationalisations of the GPCC model were disparate in details about the core practices. Sometimes, a PCC approach was encouraged in all patients without specific details, practices or examples of how this could be achieved. At other times, PCC was operationalised in fixed standardised routines used with specific practices. Using a standardised protocol when listening to patients’ narratives in conjunction with admission to the ward was an example of this standardisation. Standardised care is sometimes seen as the antithesis of PCC, i.e., it is on the opposite end of the spectrum of how patients are valued and treated in health care [46]. The question is how standard routines can be flexible enough to allow HCPs to meet the individual’s unique needs. At an organisational level, Engle et al. identified fundamental characteristics that seemed pertinent for acute inpatient care units to provide care according to evidence-based care and patient-centred care [47]. The authors identified features such as highly engaged staff where HCPs worked in teams to provide and share responsibility for care and communication with patients, with the support from leaders in their work [47]. Lydahl illustrates in the context of the individual HCP trained in a PCC approach how HCPs are inventive, continuously adjusting to combine and work around contradictory values in practice [48]. HCPs have also raised concerns about working in partnership with patients when they feel unsure about decisions taken by a patient regarding a treatment option that is not advocated as the best treatment based on best available evidence [23, 45]. Öhlen et al. argue that both individualisation and standardisation of care and routines have a place in today’s health care and should be seen as complementary rather than opposed to one another [49]. Standardisation is a way to increase and secure equity for all patients on a population-level, while individualisation will accommodate what is important to each individual patient [49].

How PCC is accomplished in health care in various practices and contexts is important knowledge to politicians, leaders, HCPs and patients throughout the health care sector. So as not to risk PCC becoming a buzzword mentioned without carefully analysing and reporting the proposed core practice regarding underlying philosophical principles and structural elements, the knowledge gap between ethical reasoning about what should be done to how it is operationalised in reality needs to become bridged [50]. We agree with other researchers that PCC is a complex concept to specify for researchers and HCPs alike [1, 9, 45, 51]. The ethical basis of PCC is considered a prerequisite for HCPs understanding of the concept of PCC [4, 5]. Nevertheless, the founders of the GPCC model are steadfast that PCC is an ethical approach that is accomplished in concrete actions [6]. In other words, it is not enough for HCPs to state that they see the person behind the patient; they also need to act so that patients and bystanders recognise that PCC have been translated into observable actions. Some of the subtleties of PCC can perhaps only be evaluated from the patient perspective, such as perceiving that HCPs have listened [4, 52], compared to more apparent observations, such as the realisation of a plan for continued care [52].

Describing PCC to its structural elements and core practices is a fundamental first step towards building a shared understanding of what it entails in practice. This step can be achieved even without common conceptualisation or terminology underlying the different schools of PCC. When studies are reported, a description of what constitutes the structural elements and what is done in health care practice (i.e., core practices) should become a priority and prerequisite [26]. Second, patients can be part of interventions and quality improvements with information about the practices conducted by giving valuable input into and validating the core practices in relation to PCC’s philosophical principles and values. Patients can comment on what core practices they perceive as helpful and to what extent they align with PCC’s philosophical principles and values. The third step involves defining and outlining the structural elements of PCC and its core practices and is a prerequisite for implementation that can increase buy-in with HCPs by facilitating their understanding of the innovation and how it relates to and differs from previous practice [23]. Increased awareness of the philosophical principles and values along with PCCs structural elements will aid HCPs in knowing what and when they can adapt certain core practices while still maintaining a PCC approach.

We believe that using the recommendations for detailing the core components of PCC described here can guide HCPs to shift between the components in the model from Fixsen et al. and reflect whether and to what extent a certain practice is in accord with the philosophical principles and values underlying the innovation.

### Methodological considerations

The case study was chosen to explore how PCC was operationalised in a real-world setting without the involvement of researchers. Conducting research in a natural environment can contribute with valuable knowledge by pointing out or targeting intuitively and functional activities chosen by the end-users, i.e., individuals with hands-on experience from their context [30]. HCPs have valuable knowledge of their work context, including the limits of what means they have available and the needs and rights of the patients they face daily, leading to realistic and beneficial practices.

We chose the recommendations of Fixsen et al. [24, 25] because the components included fitted the nature of PCC with its strong philosophical anchoring [5]. Moreover, the recommendations work well with the study’s naturalistic inquiry, where we observed the operationalisation of PCC without any in-depth information on hypothetical mechanisms of change, which is a critical aspect in programme theories [22]. However, we acknowledge that other structures to outline and detail interventions exist, such as the TIDIER checklist [43], which would have given a different presentation of PCC’s operationalisation and core components. The deductive data analysis was made in accordance with GPCC’s model as this was the model chosen and advanced by the DD change agents. We found that the model with its three structural elements was relatively straightforward when we linked the core practices to the narrative and documentation. However, the partnership was seen to contain different practices with varying foci, which is in line with the founders of the model, who see this part as central to PCC. It also points to the importance of detailing what each structural element implicates as it may include countless operationalisations. We were open to reporting emerging categories to ensure that the GPCC model and its structural elements were not used in a constraining way or forced to fit with the findings. Other approaches to report structural elements of PCC could be the use of McCormack et al.’s model [4] or previous research targeting common overarching conceptualisations of PCC such as those found by Hughes et al. [53] or Harding et al. [9]. Hughes et al. identified 10 core themes within different schools for centredness regarded to share the same underlying meaning [53]. Harding et al. reported on three large and interrelated concepts within PCC [9].

We triangulated data from three sources to gain different viewpoints for our data analysis, which is a strength of this study. However, we acknowledge a limitation of the study as no observations or interviews with patients were made to triangulate our data. Observations on site of everyday activities could have been a valuable contribution in data analysis as there may be a tendency for study participants to report aspirations rather than the actual practice of PCC [54]. Interviews with patients about their perceptions of the core practices could have given vital information about which practices were most valued and if reported core practices were in line with patients’ experiences and perceptions (e.g., did HCPs listen to what patients’ value in their care). We were surprised to find that the core practice of documenting the partnership in some units seemed to be more influenced by legal prejudices than the ethical principles and values of PCC. This result may have been lost if we had not used Fixsen et al.’s recommendations, where context is regarded as a core component. This observation reminds us how important it is to consider context in all studies directed towards operationalisation and implementation of complex innovations in the health care sector [55, 56].

## Conclusion

Our findings demonstrate the complex nature between the philosophical basis of PCC and its operationalisation in everyday care activities. Despite the study’s limitations, it reflects a unique range of operationalisations of PCC in a real-world setting in Sweden. Increased knowledge about PCC and its philosophical principles and values, contextual factors, structural elements and core practices is a prerequisite to building a common understanding of the concept. Such knowledge is essential for all stakeholders involved when PCC is operationalised as part of the implementation efforts in various health care settings.

## Supplementary Information

**Additional file 1.** Interview guide.

## Data Availability

The datasets used in this study are available from the corresponding author on reasonable request.

## Abbreviations

DD: Department for development
DI: Dyadic interview
FG: Focus group
GPCC: The Gothenburg University Centre for Person-Centred Care
HCP: Healthcare professional
PCC: Person-centred care
