# Supplementary material for: Operationalisation of person-centred care in a real-world setting: a case study with six embedded units
Source: BMC Health Serv Res. 2022 Sep 14;22:1160. doi: 10.1186/s12913-022-08516-y (PMC9476689; doi:10.1186/s12913-022-08516-y)
Supplement: Supplementary file 1 — Additional file 1. Interview guide. [file 12913_2022_8516_MOESM1_ESM.docx]

**Additional file 1. Interview guide**

**Interview guide: Interviews with change agents at the department for development and six health care units (English*)**

*This is a translation from Swedish, provided for publication reasons and might not present a validated translation.

You have been asked to participate in this interview as you have been identified as one of the professionals who hold or have been given a strategic role in driving the change for more person-centred care at the Region or at your unit.

**Presentation of the research group:**

Introduction of ourselves and the project. Previous experience in implementation science, research, work in clinical care, and current positions as a Doctoral student, professor and assistant professor at Dalarna University.

Project background: As of today, knowledge of how organisations and leaders can support implementation of person-centred care effectively is lacking. The interview today is part of a large project aimed at increasing understanding of the processes that take place in a health care context when new knowledge is implemented in an organisation. We want to understand what happens in a natural setting when person-centred care is encouraged by people in the organisation and not orchestrated by researchers. What kind of strategies or activities are used to enable more person-centred care across a health care context and what are the effects of the chosen strategies? We follow the department of development and six units at the Region, and you are part of one of them. We collect data in many different forms and today we are conducting interviews.

**Introduction about the group discussion:**

The dialogue from the interview will be recorded and then transferred to a text file. All information from this interview is kept in a locked location at Dalarna University. Only people in the research group will have access to this information. When we report the results from this project no single person will be able to be identified and you will be able to validate the results before they are published as an article. We are interested in all your thoughts regarding our interview questions and there are no right or wrong answers. The interview is not a test of your knowledge on implementation or person-centred care. If there are things that you say that we don’t quite understand we will ask you to explain what you mean so that we are sure to interpret your input and discussions correctly.

Questions:

1. Can we start off with a round where you tell me your first name, your profession or role in supporting more person-centred care at your workplace?
2. Now lets talk about the changes you are carrying out to make care at your workplace more person-centred? Please tell med about the changes that you have made and plan to make. Prompts (if needed): How do you connect this activity to person-centred care?
3. Summarise the discussions about changed work routines to make care more person-centred and then ask: is there anything that we have missed that you think is important to add? Please tell me.
4. Now let’s move on to talking about the activities or strategies that you are carrying out to support more person-centred care at your workplace. Please tell me about the activities or actions that you are using to bring about more person-centred care?

Prompts (if needed): for example, activities directed towards health care professionals, towards the organisation or perhaps towards the environment i.e., such as physical environment or medical records.

1. What kind of change do you think the activities will affect?

Prompts (if needed): for example, increased knowledge, improved cooperation…

1. In what way do you think these activities [give example of activities mentioned by participants] will support the change to more person-centred care in the Region?
2. What do you think are the most important enabling factors to support more person-centred care?

Why do you think these are enabling factors? What do you base your thoughts on? How do you work to increase enabling factors?

1. What are the most important barriers to working more person-centred?

Why do you think these are hindering factors? What do you base your thoughts on? How do you tackle barriers?

1. What activities have you carried out so far? Have you made any changes in your strategy or activities up until today? Why?
2. Summarise the discussions about strategies and activities and then ask: is there anything that we have missed that you think is important to add? Please tell me.

Prompts:

Can you tell me more?

Can you give me an example?

Do you have anything more to add?

Do you mean that…… [moderator summarises the content of the discussion]?

Does that mean…… [moderator summarises the content of the discussion]?

How do you realise … (if participants relate changes in care related to abstract concepts e.g., partnership) in practice and work routines?

If I try to summarise/interpret what you just discussed…… [moderator summarises the content of the discussion]? Did I get it right?
